# Identification of growth stage-specific watering thresholds for drought screening in *Solanum aethiopicum* Shum

**DOI:** 10.1038/s41598-020-58035-1

**Published:** 2020-01-21

**Authors:** Mildred Julian Nakanwagi, Godfrey Sseremba, Nahamya Pamela Kabod, Michael Masanza, Elizabeth Balyejusa Kizito

**Affiliations:** 1 Department of Agricultural and Biological Sciences, Faculty of Science and Technology, Uganda Christian University, P.O. Box 4 Mukono, Uganda; 2 National Coffee Research Institute, National Agricultural Research Organization, P.O. Box 185 Mukono, Uganda

**Keywords:** Plant physiology, Drought

## Abstract

Effective phenotyping for drought resistance is a pre-requisite for identification of modest crop varieties for farmers. For neglected and underutilized crops such as *Solanum aethiopicum* Shum group, no drought screening protocol based on rigorous iterations has been documented. A split-plot nested treatment structure was arranged in an experiment to identify growth stage-specific watering thresholds for this crop. Three plant growth stages (main plot; seedling, vegetative and flowering), watering regime at plant growth stage (2 regimes; well-watered and drought stressed) and day since last watering at plant growth stage were evaluated for soil moisture content (SMC), leaf wilting score (LWS), number of green leaves per plant (LPP) and leaf blade width (LBW). Highly significant differences (p < 0.001) were found at the different plant growth stages, watering regime (WR) within plant growth stage, and day within WR and plant growth stage. Under drought stress treatment, SMC declined exponentially at each stage. The earliest leaf wilting, reduction in LPP and LBW were generally observed at flowering followed by vegetative and slowest at the seedling stage. For future effective drought phenotyping studies in *S*. *aethiopicum* Shum and related crops, we recommend setting minimum drought stress treatments below 18% SMC at which the LWS is ≥2 at the vegetative.

## Introduction

*Solanum aethiopicum* Shum is a nutrient rich leafy vegetable consumed in Sub Saharan Africa^1^. It is thus a potential remedial to hidden hunger^2–8^. Smallholder farmers and vendors find the crop a largely affordable enterprise because of low input requirements along the production-marketing value chain^1,9^. In Uganda only, at least 4,000,000 people are directly engaged in either production or vending of the *S*. *aethiopicum* Shum^1,10,11^; justifying need for research investment. High population involvement has as well been reported elsewhere^3,7,12–14^. Extent of contribution of the crop to alleviation of hidden hunger is however, threatened by biotic and abiotic constraints^15–17^. Postharvest deterioration and drought stress are the two major moisture-related abiotic challenges directly affecting market vendors and farmers, respectively^1,16,18^. At the market place, non-turgid leaves either sell for low price or they are entirely rejected by consumers, attracting a direct loss to the vendors^9,18^. Similarly, drought stress reduces leaf size thereby reducing the harvest index^19,20^ to the detriment of the farmer. These issues point to a need for identification of long storage and/or drought tolerant varieties^1,2,9^. However, *S*. *aethiopicum* is a research neglected crop^5^ and inadequate breeding clues for drought improvement exist^17,20–22^.

An absence of a reliable drought phenotyping protocol for *S*. *aethiopicum* Shum group curtails the development of improved varieties^23^. Optimum drought screening conditions in terms of effective crop growth stage for selection against drought stress, wilting points and discerning parameters had not been thoroughly investigated. Studies by Sseremba *et al*.^17^, Banik *et al*.^24^, and Kesiime *et al*.^25^; as well as reviews by Osakabe *et al*.^26^ and Fahad *et al*.^27^ opined that parameters namely leaf relative water content (LRWC), plant height and number of green leaves per plant can discriminate among genotypes under drought stress at 25 percent field capacity. Though, comparisons across growth stage and continuous moisture depletion were not made. Whereas the LRWC is one of the most important measure of plant-water status^17,23,24^ in leafy vegetables as an indicator of leaf quality^17,28,29^, the parameter is semi-destructive^24,25^ and too time-consuming to be used in high throughput and large scale phenotyping. The current study took care of three growth stages namely seedling, vegetative and reproductive whereby watering was withheld permanently at each stage. Further, non-destructive sensor-based monitoring of soil moisture content, chlorophyll content and stomatal conductance was made; in addition to leaf wilting scores, leaf size^19,30^ and leaf number^27^.

It is reported that watering depletion impairs growth as a result of decreased cell turgor and leaf wilting, chlorophyll content, stomatal conductance for CO_2_/O_2_ and reduced photosynthetic efficiency^23,30^. The reduction in photosynthetic efficiency is an intermediate effect through a signal^31,32^. Either an abscisic acid (ABA) dependent or ABA independent signal in a feedback mechanism of relative soil-plant water tension^32–36^, stimulates stomata closure (reduces gas exchange) in order to prevent further water loss. Otherwise, a continued gas exchange that maximizes photosynthetic efficiency under drought stress results in leaf rolling (wilting)^23,37^. Closed stomata attract exhaustion in levels of CO_2_ for photosynthesis at the expense of photorespiration (due to accumulated O_2_ relative to CO_2_)^19,26,31,32^. Photorespiration produces reactive oxygen species which damage the thylakoids; thereby reducing the chlorophyll content. The devastation in photosynthetic apparatus and thus reduced cell division and dry matter accumulation continues to worsen till plant death unless re-watering^29,30^ is done.

As mentioned already, consequent to compromised photosynthetic efficiency, drought effects in leafy vegetables are commonly morphologically manifested as plant stunting, reduced leaf size, reduced number of leaves, reduced yield and leaf wilting/rolling^27^. As a plant grows, adaptive structures such as extensive root system and waxing of leaf surfaces (cell wall remodeling) also develop^38^. Vegetative stage in grain crops is however, less critical to drought than flowering/reproductive stage in almost all flowering plants.

In leafy vegetables, it is the vegetative stage that matters most to growers and severe drought affects such crops^18,27,28^. This study took interest in verifying water depletion effects on leaf wilting, number of green leaves per plant and leaf blade width in *S*. *aethiopicum* Shum. The main objective of the study was therefore to identify growth stage-specific watering thresholds in *S*. *aethiopicum* Shum and specifically to determine the gradual tendency of leaf morphological traits amidst declining soil moisture.

## Results

### Effect of watering depletion on leaf traits

There was a very highly significant difference (p < 0.001) in stages, watering regime by plant growth stage and day since last watering by plant growth stage for all the parameters measured (SMC, LWS, LPP and LBW) (Table 1). Under drought stress (DS) over time (days since last watering), seedling stage had the wettest soil (21.2% SMC) followed by vegetative (20.6%) and the least was under flowering stage (11.8% SMC) (Table 2).

**Table 1:** Mean squares for soil moisture content and leaf traits measured.

Source of variation	d.f	SMC	LWS	LPP	LBW
Stage	2	15212.2***	52.2***	46239.9***	986.6***
Stage/WR	3	341719.5***	741.7***	14582.8***	1910.2***
Stage/WR/Day	60	8720.2***	52.4***	1010.5***	455.7***
Residual	5202	22.6	0.2	31.3	5.9

***Significance at 0.001 error margin; WR, watering regime; SMC, soil moisture content; LWS, leaf wilting score; LPP, leaves per plant; LBW, leaf blade width.

**Table 2:** Mean soil moisture content at different growth stages over time.

Day	Seedling		Vegetative		Flowering		s.e.d and l.s.d for mean comparison		
	WW	DS	WW	DS	WW	DS	category	s.e.d	l.s.d (α = 1%)
1	50.0	50.0	47.7	46.5	45.2	45.1	Stage	0.16	0.42
2	47.2	46.1	47.7	46.5	45.3	17.2	Stage/WR	0.23	0.60
3	48.3	39.6	45.5	21.5	45.8	14.1	Stage/WR/Day	0.75	1.93
4	48.6	36.0	42.6	16.1	45.5	12.0			
5	47.6	32.3	45.8	12.9	45.1	9.4			
6	46.5	22.7	45.1	9.2	45.0	8.7			
7	46.9	18.0	44.8	5.8	44.7	4.2			
8	47.5	15.3	45.0	4.7	44.8	3.3			
9	46.0	14.8			44.9	2.6			
10	46.4	9.9			44.4	1.6			
11	45.3	8.6							
12	45.6	7.7							
13	45.2	6.9							
14	45.1	6.2							
15	45.5	4.3							
**Mean**	**46.8**	**21.2**	**45.5**	**20.6**	**45.1**	**11.8**			

WR, watering regime; WW, well-watered; DS, drought stressed; s.e.d, standard error of difference; l.s.d, leaf significant difference.

Across days, the highest LWS under DS was observed at flowering stage (2.6) followed by vegetative (2.4), and seedling stage had the lowest score (2.0) (Table 3). At seedling stage, leaf wilting was visible after day 6. For both vegetative and flowering stages, leaf wilting was visible after day 2. The differences between LWS from date of visible leaf wilting to the extreme LWS attained was lower for the flowering stage (difference of 3.3 attained on the 10^th^ day since stress imposition) than for the vegetative stage (difference of 3.5 attained on the 8^th^ day since stress imposition). The extreme LWS observed was 3.9 on 15^th^ day followed by 4.3 on the 10^th^ day and 4.5 on the 8^th^ since stress imposition for seedling, flowering and vegetative stages, respectively. As expected, the LWS under WW was always 1.0 (no leaf wilting).

**Table 3:** Mean leaf wilting scores at different growth stages over time.

Day	Seedling		Vegetative		Flowering		s.e.d and l.s.d for mean comparison		
	WW	DS	WW	DS	WW	DS	category	s.e.d	l.s.d (α = 1%)
1	1.0	1.0	1.0	1.0	1.0	1.0	Stage	0.017	0.043
2	1.0	1.0	1.0	1.0	1.0	1.0	Stage/WR	0.023	0.060
3	1.0	1.0	1.0	1.2	1.0	1.8	Stage/WR/Day	0.075	0.194
4	1.0	1.0	1.0	1.4	1.0	2.5			
5	1.0	1.0	1.0	2.8	1.0	2.4			
6	1.0	1.0	1.0	3.3	1.0	2.9			
7	1.0	1.8	1.0	4.3	1.0	3.1			
8	1.0	1.8	1.0	4.5	1.0	3.1			
9	1.0	1.8			1.0	4.0			
10	1.0	2.9			1.0	4.3			
11	1.0	3.0							
12	1.0	2.9							
13	1.0	3.0							
14	1.0	2.5							
15	1.0	3.9							
**Mean**	**1.0**	**2.0**	**1.0**	**2.4**	**1.0**	**2.6**			

WR, watering regime; WW, well-watered; DS, drought stressed; s.e.d, standard error of difference; l.s.d, leaf significant difference.

Number of green leaves per plant (LPP) under DS were highest (LPP = 12) at flowering followed by seedling (4) and vegetative (4) (Table 4). At seeding, vegetative and flowering, a reduction in LPP was observed after day 13, day 3 and day 3, respectively. Under well watering (WW) regime, LPP was always higher than that under DS at each stage of plant growth.

**Table 4:** Mean number of green leaves per plant at different growth stages over time.

Day	Seedling		Vegetative		Flowering		s.e.d and l.s.d for mean comparison		
	WW	DS	WW	DS	WW	DS	category	s.e.d	l.s.d (α = 1%)
1	3	3	5	5	12	14	Stage	0.2	0.5
2	3	4	5	5	18	17	Stage/WR	0.3	0.7
3	4	4	5	5	18	18	Stage/WR/Day	0.9	2.3
4	4	4	6	5	18	11			
5	5	4	7	4	20	10			
6	6	4	11	3	17	8			
7	6	4	7	3	23	11			
8	8	4	13	1	22	10			
9	9	4			24	10			
10	13	4			21	9			
11	10	4							
12	15	4							
13	17	4							
14	19	3							
15	20	3							
**Mean**	**9**	**4**	**8**	**4**	**19**	**12**			

WR, watering regime; WW, well-watered; DS, drought stressed; s.e.d, standard error of difference; l.s.d, leaf significant difference.

As naturally may be expected, LBW was highest at flowering stage (10.8 cm) followed by vegetative (10.0 cm) and then seedling stage (8.9 cm) (Table 5). Within plant growth stage, LBW started declining after day 6 for the seedling stage. For vegetative and flowering stages, the decline in LBW was observed after day 3 and day 2, respectively (the measurement was taken on most fully open but non-wilted leaves).

**Table 5:** Mean leaf blade width over time nested within watering regime nested within growth stage.

Day	Seedling		Vegetative		Flowering		s.e.d and l.s.d for mean comparison		
	WW	DS	WW	DS	WW	DS	category	s.e.d	l.s.d (α = 1%)
1	4.8	4.3	9.4	10.5	13.6	13.7	Stage	0.08	0.21
2	4.9	6.6	9.4	10.5	13.4	12.9	Stage/WR	0.12	0.31
3	7.5	7.1	11.8	10.9	12.5	11.7	Stage/WR/Day	0.38	0.99
4	9.6	9.5	12.4	10.7	12.6	10.9			
5	10.6	9.7	13.8	9.9	14.2	12.7			
6	13.1	11.8	13.7	9.9	12.7	12.6			
7	13.2	11.8	14.2	9.1	10.5	8.1			
8	15.0	11.4	13.4	8.8	10.3	8.2			
9	10.4	9.1			9.2	8.4			
10	14.7	10.0			11.9	8.5			
11	16.4	12.2							
12	13.3	8.3							
13	12.7	8.3							
14	11.9	7.0							
15	11.2	6.6							
**Mean**	**11.3**	**8.9**	**12.3**	**10.0**	**12.1**	**10.8**			

WR, watering regime; WW, well-watered; DS, drought stressed; s.e.d, standard error of difference; l.s.d, leaf significant difference.

### Stage-specific watering thresholds

#### Moisture decline over time at each stage

**Figure 1:**
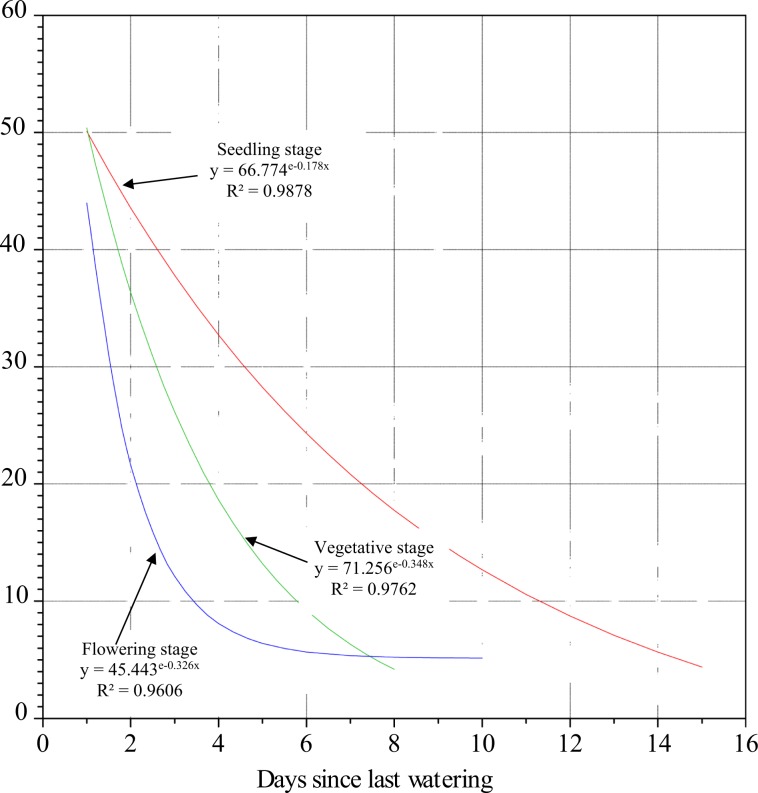
Soil moisture depletion curve at seedling, vegetative and flowering stages during the experiment.

Standard curve for seedling stage was above that for vegetative followed lastly by that for flowering stage (Fig. 1). The moisture decline was steepest for flowering stage followed by vegetative and relatively gentle for seedling stage. At flowering stage, within two days of drought stress, the soil moisture content (SMC) dropped from 45.2% to 17.2% and it continued to drop to 3.3% by the 8^th^ day and continued to decline to 1.6% by day 10. For the vegetative stage, SMC declined quite fast from 46.5% (day 1) to 4% by day 8. The SMC declined the slowest from a high level (50.0%) at day 1 to 15.3% (day 8); then the moisture continued with the slow decline to 4.3% at ~day 15. The coefficients of determination (R^2^) were very high for both seedling (98.9%) and vegetative (97.6%), and high for flowering (96.1%) stages based on exponential models.

#### Leaf wilting point at each growth stage

**Figure 2:**
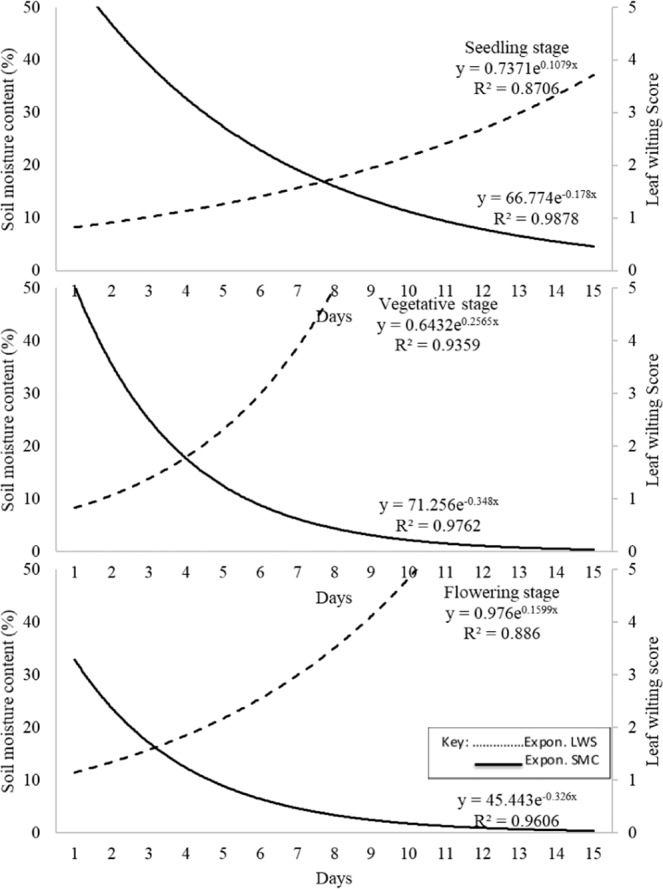
Leaf wilting curves for seedling, vegetative and flowering stages under declining soil moisture content over time.

The standard curves touched the SMC curve at critical wilting points below whose moisture levels leaf wilting causes economic loss. The seedling, vegetative and flowering stage curves touched the SMC curve at 16.5, 18 and 16 percent moisture levels respectively (Fig. 2). Clearly visible leaf wilting symptoms (LWS ≥ 2.0) occurred at 20% SMC by day 8, 4 and 3 for plants at seedling, vegetative and flowering stages, respectively. The highest leaf wilting score was attained at SMC 9% (seedling), 8% (vegetative) and 11% (flowering stage) by the 15^th^, 8^th^ and 10^th^ day. The generated standard curves were exponential with R^2^ = 0.87, 0.97 and 0.96 for seedling, vegetative and flowering stages, respectively.

#### Critical soil moisture content for preventing leaf loss

**Figure 3:**
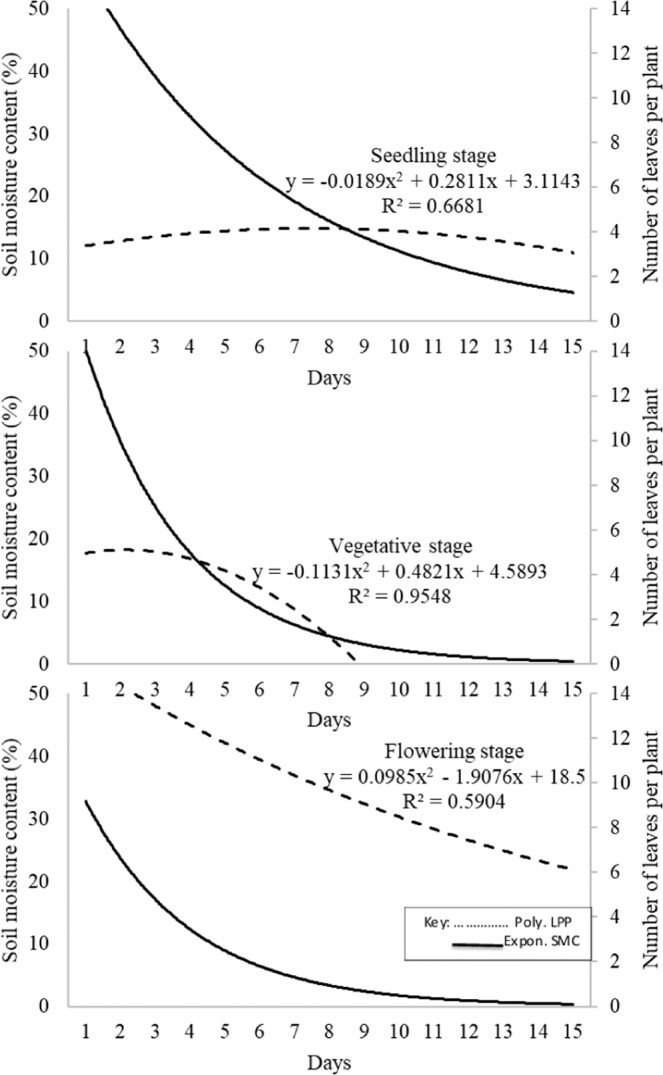
Standard curves for leaves per plant and soil moisture content at three growth stages over time.

Generally, the leaf loss curve at flowering was above that for vegetative followed by that for seedling stage (Fig. 3). During seedling stage, LPP were stable from ~46.0% SMC up to 15% SMC. However, the curve for seedling exhibited a relatively gradual decrease in number of leaves after 8^th^ day. At vegetative stage, LPP began gradually reduce after the 3^rd^ day while a fast decrease in number of leaves was observed after the 4^th^ day when the SMC was 16%. On withholding water, LPP started reducing after a day however the decrease was gradual. Further, early decline in SMC was detectable through corresponding reductions in LPP for both vegetative and flowering stages. When SMC decline continued beyond 16%, it was clear that all leaves could most likely be lost under vegetative followed by seedling and the highest leaf retention (LPP at extreme moisture deficit) was observed at flowering stage. The generated standard curves were polynomial with R^2^ = 0.67, 0.96 and 0.59 for seedling, vegetative and flowering stages, respectively.

#### Critical soil moisture content for preventing shrinkage in leaf blade width

**Figure 4:**
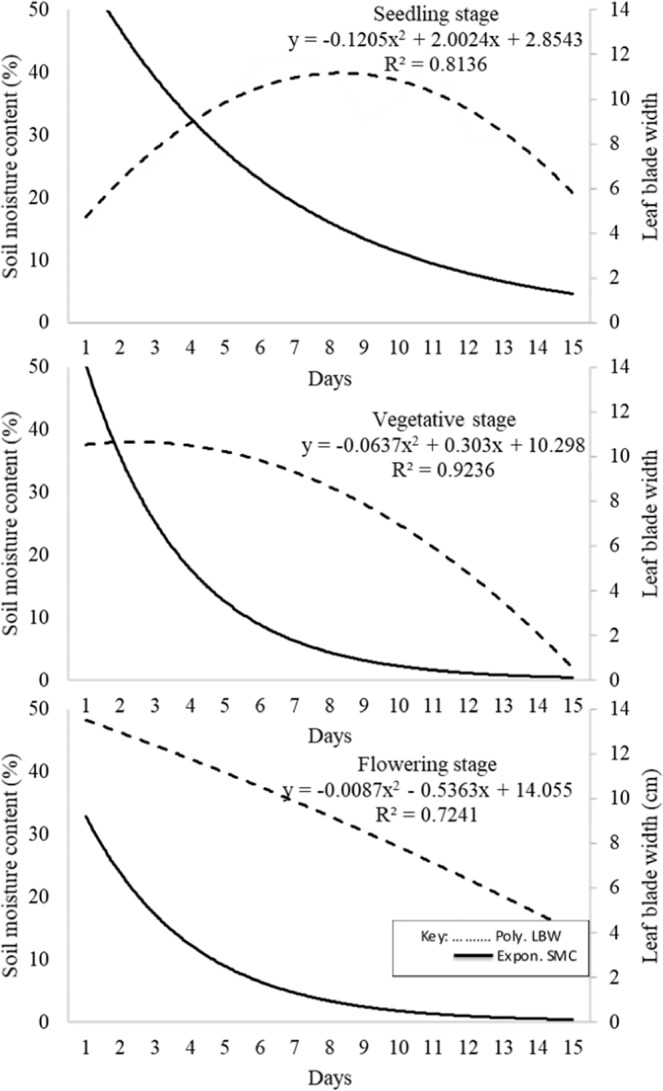
Standard curves for leaf blade width and soil moisture content at seedling, vegetative and flowering stages over time.

Standard curve at flowering was above that for vegetative below which followed the seedling stage curve (Fig. 4). At seedling and vegetative the critical SMC were at 32% by the 4^th^ day and 38% by the 2.5^th^ day. The critical SMC for flowering stage could not be attained in number of days stipulated for drought stress. Below the critical SMC levels, LBW started declining. A gradual increase in LBW was observed from day 1 to day 8 at SMC 40% at seedling stage beyond which the LBW size begun a gradually decrease. At vegetative stage, leaf expansion was observed between the 1^st^ and 2^nd^ day after which a gradual decline was observed until the 8^th^ day. The generated standard curves were polynomial with R^2^ = 0.81, 0.92 and 0.72 for seedling, vegetative and flowering stages, respectively.

## Discussion

Watering depletion gradually led to decline in SMC which correspondingly increased leaf wilting, reduced the number of green leaves per plant and reduced the leaf size (measured as leaf blade with). Fastest leaf wilting, drop in LPP and LBW at flowering than rest of stages points to high plant sensitivity at flowering^23^. The view of high plant sensitivity to moisture changes at flowering has severally been corroborated in grain crops such as maize^19,26–28^. Overall, crop moisture requirements increase with growth stage^23,26^. At seedling stage, moisture deficit can lead to complete crop failure when germination and emergence is affected^27^. In this study however, drought stress was imposed after seedling emergence and so, germination/emergence was studied. The quantities of water required at seedling stage are way below that at subsequent stages^27,35,39^. This study reported of high SMC at seedling as compared to other stages. The reason is that seedlings do not have the capacity to take up and transpire large quantities of moisture from soil. The main avenue of soil moisture loss at seedling stage can be direct evaporation from the soil surface depending on solar radiation, ambient temperature and relative humidity^19,27,35,39^. Indeed, when watering was withheld, it took about twice the period as compared to vegetative and seedling stages for soil moisture to drop to same level across stages. Specifically, whereas SMC decline from field capacity to 4.5% for the case of vegetative stage in 8 days and flowering stages required 10 days, the same reduction at seedling stage had taken 15 days. The suggestion is that it would be expensive in terms of time required to observe selectable variation. Focusing on rest of stages (vegetative and flowering), a bigger difference in visible leaf wilting for a shorter period was observed for vegetative than the flowering stage (Table 5). In addition, the highest coefficient of correlation was observed at vegetative stage for all evaluated traits. It implies that screening for drought tolerance at vegetative stage is achievable in the shortest period. Being a crop grown mainly for its leaves at the vegetative stage^1^, *S*. *aethiopicum* Shum variety selection at the harvest could result in direct benefits to most of value chain actors such as farmers, vendors and consumers^9^.

The *S*. a*ethiopicum* Shum group seedlings, just like for other crops, require constant watering; lest they quickly become wilted and productivity declines as observed earlier in *Miscanthus* spp. by^38^. This study observed that the critical SMC was highest at vegetative followed by seedling and flowering. However, response to water deficit stress was earliest for flowering followed by vegetative and seedling stages. Nonetheless, the plants at flowering stage endured for the longest (without complete leaf wilting) compared to the vegetative stage; suggesting suitability of the latter for drought screening. As seedling stage, plants do not demand a lot of water and there is limited surface area for water loss through transpiration^23^. Conversely, at advanced growth stages (typically the reproductive), plant structure is well developed to withstand low moisture tension of the soil^19^. At the flowering stage, plant systems are tuned to survival fitness through maximizing flowering/pollination success/fertilization success and subsequent seed development^19,23^. During the flowering period, plants express drought stress detection genes for “housekeeping” to safeguard against excessive water loss^26^; this explains tendency of leaves to wilt at high soil moisture potential^23,26^ even though complete leaf death is delayed. The potential defensive “housekeeping” against drought stress effects at flowering stage also accounts for the reduction in LPP and LBW at relatively high soil moisture potential^26,31,32^. There exists a clear difference in critical SMC at seedling, vegetative as compared to flowering stage.

A reduction in SMC below a particular critical level results in manifestation of drought effects^24,35,39^. For maximum productivity of the crop, it is logical to apply stage-specific optima of water which do not fall below critical levels^19,27^. Based on LWS, the critical watering intervals (action threshold for watering after a period of drought stress) should be eight, four and three days for seedling, vegetative and flowering stages, respectively. The critical watering intervals based on LWS were the same as those for LPP and LBW for the case of seedling and vegetative stages. This study could not establish the action thresholds for watering at flowering stage based on LPP and LBW since the values of the parameters never shrunk to extreme low levels as SMC declined. This study did not also delve into economic thresholds for watering requirements for *S*. *aethiopicum* Shum. During drought phenotyping of germplasm, drought stress treatments in *S*. *aethiopicum* Shum group should be set below the identified critical levels. An actual SMC to impose as drought stress treatment would be guided by the diversity of test germplasm^23,28,35,37^; and the contextual breeding objective in terms of parameters to measure (say, LWS, LPP or LBW) and desired selection intensity. In this study, focus was on three morphological variables (LWS, LPP and leaf size); raising need to corroborate findings when physiological traits as well as molecular underpinnings are also included^33,34^.

## Materials and Methods

### Plant material

A farmer preferred genotype E16 (also coded as 184 P, pedigree SAS184/P/2015)^17,20–22,29^ was selected following an earlier on-farm participatory evaluation (results of farmer preference study are unpublished). The genotype E16^21^ out of ten *S. aethiopicum* Shum group genotypes was advanced for national performance trial for yield evaluation, and distinctiveness, uniformity and stability (DUS) testing by Uganda Christian University. The farmer desirability of E16 and its relative morphological DUS prompted us to adopt it as a model accession for drought phenotyping.

### Study location

**Figure 5:**
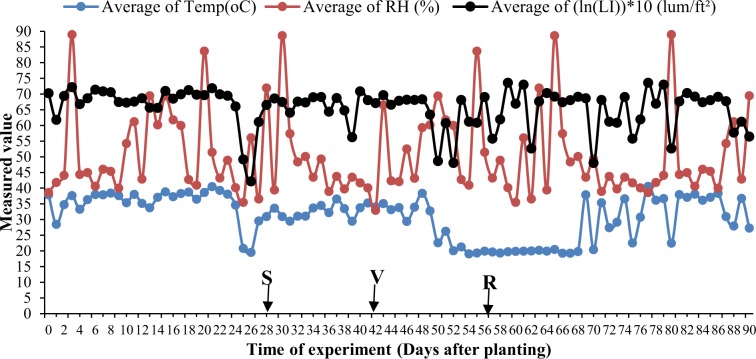
Variation in mid-day temperature, relative humidity and light intensity during period of the experiment. S, V and R; stages at which watering depletion was imposed (S, seedling stage; V, vegetative stage; R, reproductive stage). Temp, temperature; RH, relative humidity; LI, light intensity; ln(LI)*10; natural logarithm (ln) of light intensity (LI) multiplied by a constant 10.

An experiment was carried out in screen house at Uganda Christian University, Mukono for 90 days. Screen house weather conditions. Midday screen house temperature (°C), relative humidity (%) and light intensity (lum/ft^2^) remained relatively stable (slight variations) throughout the period of the study (Fig. 5). The model screen house temperature (Temp), relative humidity (RH) and light intensity (LI) was 38 °C, 38.6% and 48.0 (122.0) lum/ft^2^, respectively (Table 6). The weather data was recorded using a data logger (LGR S/N: 10280023, SEN S/N: 10280023).

**Table 6:** Summary of screen house weather data during the experiment.

Statistic	Temp (oC)	RH (%)	LI (lum/ft²)	
			Untransformed data [LI]	Transformed data [(ln(LI))*10]
Mode	38.0	38.6	122.0	48.0
Mean	31.1	50.5	820.6	65.6
Median	33.7	44.4	876.4	67.8
St. Dev.	7.1	13.5	348.5	6.3

Temp, temperature; RH, relative humidity; LI, light intensity; ln(LI)*10; natural logarithm (ln) of light intensity (LI) multiplied by a constant 10; St. Dev., standard deviation.

### Experimental design

A split-plot arrangement was visualized in a nested treatment structure. The main plot and subplot factors were watering regime (WR) and day since last watering by plant growth stage (Day), respectively. Two WRs namely well-watering (WW) and drought stress (DR); and three growth stages (seedling, vegetative and flowering) were evaluated. The seedling, vegetative and flowering stages were defined as 4 weeks after planting (WAP), 6 WAP and 8 WAP, respectively. For all stages, planting was carried out the same day in December 2017 (12^th^ December 2017).

For each stage, 20 pots (filled with a potting substrate comprising a mixture of loam soil and manure in a ratio 3: 1) were allocated per replication. The pots were of 10 kg soil capacity. Field capacity (100% FC) of the potting substrate was established following a procedure described in Sseremba *et al*.^17^ and Nakanwagi *et al*.^20^. It was found to be ~3.0 litres of water per 10 kg of potting substrate. Therefore, the WW regime was at 100% FC.

Two replications per WR per stage were used. Each pot was planted with four directly sowed seedlings which were thinned to two plants per pot at three weeks after planting. The replication was not taken as a factor (no blocking) and the experiment was conducted in the screen house at Department of Agricultural and Biological Sciences, Uganda Christian University. The actual drought treatment experiment lasted 15, 8 and 10 days for the seedling, vegetative and flowering stages, respectively.

In effect, at each plant growth stage and WR, 80 plants were evaluated. The DS regime was introduced following a WW pre-watering until the respective evaluation stage. That is to say, experimental pots were continuously well-watered until they reached designated growth stages at which watering was withheld (hence DS). The WW controls and DS experimental pots were then maintained throughout the study. Lighting, temperature and humidity conditions of the screen house used as well as agronomic practices for potted plants were similar to what was described in our earlier study^29^ and as updated using the weather data presented in Fig. 5 and Table 6.

### Data collection

Data was collected at seedling, vegetative and vegetative stages starting from day 1 since withholding of watering until severe leaf wilting symptoms become apparent. Individual pots were the observational unit for the case of soil moisture content (SMC). The leaf morphological traits namely leaf wilting score (LWS), number of green leaves per plant (LPP) and leaf blade width (LBW) were measured on individual plants. Decision to concentrate on the four parameters was based on the insights provided by Banik *et al*.^24^ and Sseremba *et al*.^17,29^. The SMC was measured in individual pots in percentage volume by volume using a portable digital soil moisture meter (model SKZ111K-1B, SKZ Industrial Co., Limited, D-11, No.9 Lanxiang road, Tianqiao District, Jinan, Shandong province, China). The LWS was taken on individual plants using a scale of 1 to 5 where 1 = no visible leaf wilting symptoms, 2 = 1–25% of leaves are showing slight wilting; 3 = 25–50% of leaves are wilted; 4 = 50–100% of leaves are wilted but still attached to plant; and 5 = complete defoliation and stem scorching. The LPP and LBW are measured as described in Sseremba *et al*.^17^.

### Statistical analysis

#### Effect of watering depletion on leaf traits

A nested design model was analyzed using GenStat 12^th^ edition (VSN International, Hemel Hempstead, UK). At experimental setup, the number of days since last watering (Day) was nested within watering regime (WR) and plant growth stage (Stage). The following analysis of variance model was thus considered:

$${Y}_{ijk}=\mu +Stag{e}_{i}+Stag{e}_{i}(W{L}_{j}(Da{y}_{k}))+{\varepsilon }_{ijk}$$

where ${Y}_{ijk}$ stands for observed measurement (any of SMC, LWS, LPP and LBW); $\mu$ stands for grand mean; and ${\varepsilon }_{ijk}$ stands for random error. $Stag{e}_{i}$, $W{L}_{j}$ and $Da{y}_{k}$ stand for the ${i}^{th}$ plant growth stage, ${j}^{th}$ watering regime nested within growth stage, and ${k}^{th}$ day since last watering nested within watering regime and growth stage. Differences (least significant differences) among treatments were declared at 1% error margin.

#### Identifying stage-specific watering thresholds

Taking growth stage as a grouping factor, a non-linear exponential regression model of form $Y=a+b{r}^{X}$ was analyzed using GenStat Release 12.1. The $Y$ and $X$ standard for response and explanatory variates; while *a*, *b* and *r* are non-linear regression parameters. The *r* parameter is elaborated as $r={e}^{-k}$; that is to say, $\,k=-\,lo{g}_{e}r$. Hence, the exponential model can as well be expressed as $Y=a+b{e}^{-kX}$. The regression model for each pair of variates (Table 7) was considered significant at 5% error margin. Alongside exponential regressions in GenStat, iterations for alternative models (linear, logarithmic, polynomial and power) were conducted in spreadsheets using Pivot Chart with a view of identifying a model for each pair of variates where mean squares (MS) are minimized. Minimum MS across model iterations was judged based on proportion of variance explained (R^2^). A model resulting in maximum R^2^ was selected and appended on the standard curve generated from the exponential model $Y=a+b{r}^{X}$.

**Table 7:** Model variate pairs for moisture depletion related questions in *S*. *aethiopicum* Shum.

Response (Y) variate	Explanatory (X) variate	Test question
Soil moisture content (SMC)	Days since last watering	How different is SMC decline over time at each growth stage?
Leaf wilting score	SMC	What is the wilting point at each growth stage?
Leaves per plant (LPP)	SMC	At which SMC does LPP begin to decline for each growth stage?
Leaf blade length (LBL)	SMC	At which SMC does LBL begin to decline for each growth stage?
Leaf blade width (LBW)	SMC	At which SMC does LBW begin to decline for each growth stage?

## Conclusion

Watering depletion negatively affects leaf morphological traits in *S. aethiopicum* Shum group. The plants continue to wilt, lose leaves and reduce leaf size as long as the water deficit treatment is sustained. The leaf wilting point at seedling, vegetative and flowering stage were identified as 16.5%, 18% and 16% SMC, respectively. Visible leaf wilting symptoms (LWS ≥ 2.0) for seedling, vegetative and flowering stages can be observed by the 8^th^, 4^th^ and 3^rd^ day respectively. We decline to conclude on watering intervals required to prevent soil moisture from dropping to below wilting points or visible leaf wilting symptoms at each stage because of likely seasonal differences in ambient temperature and relative humidity which might influence rates of evapotranspiration; an issue for further research. Since observable leaf wilting symptoms generally occur at SMC levels below those for drop in LPP and LBW, drought screening treatments should include at least a treatment of <20% SMC for the evaluated stages. These findings are useful guide for drought phenotyping of germplasm in *S*. *aethiopicum* Shum as well as related species.

## References

[1] PAEPARD. Better vegetables better lives: Improving African indigenous varieties for greater nutrition and livelihoods (2018).

[2] Bisamaza M, Banadda N (2017). Solar drying and sun drying as processing techniques to enhance the availability of selected African indigenous vegetables, Solanum aethiopicum and Amaranthus lividus for nutrition and food security in Uganda. African Journal of Food Science and Technology.

[3] Cernansky R (2015). Super vegetables. Nature.

[4] Chinedu SN (2011). Proximate and phytochemical analyses of Solanum aethiopicum L. and Solanum macrocarpon L. fruits. Research Journal of Chemical Sciences.

[5] Padulosi, S., Thompson, J. & Rudebjer, P. *Fighting poverty, hunger and malnutrition with neglected and underutilized species: needs, challenges and the way forward*. (Bioversity International, 2013).

[6] Pincus, L. M. *Increasing indigenous vegetable yield and nutritional quality through traditionally-and scientifically-informed soil fertility management*. (University of California, Davis, 2015).

[7] Stone, A. *et al*. *Africa’s indigenous crops: State of the world 2011*. (Worldwatch Institute, 2011).

[8] Von Grebmer, K. *et al*. *2015 Global hunger index: Armed conflict and the challenge of hunger*. (Intl Food Policy Res Inst, 2015).

[9] USAID. *The fresh fruit and vegetable markets of East Africa: an assessment of regional value chain actors, activities and constraints in Kenya, Tanzania and Uganda*. 1–87 (United States Agency for International Development (USAID), 2013).

[10] Sseremba G (2017). Diversity and distribution of African indigenous vegetable species in Uganda. International Journal of Biodiversity and Conservation.

[11] UBOS. *Uganda National Household Survey 2016/17*. 1–272 (Uganda Bureau of Statistics (UBOS), Government of Uganda, 2017).

[12] Abukutsa-Onyango, M. O., Adipala, E., Tusiime, G., Majaliwa, J. G. M. & others. Strategic repositioning of African indigenous vegetables in the Horticulture Sector. in *Second RUFORUM Biennial Regional Conference on‘ Building capacity for food security in Africa’, Entebbe, Uganda, 20–24 September 2010* 1413–1419 (RUFORUM, 2010).

[13] Ebert A (2014). Potential of underutilized traditional vegetables and legume crops to contribute to food and nutritional security, income and more sustainable production systems. Sustainability.

[14] Omulo, D. Value addition on traditional vegetables: an impact assessment on women farmers in Lugari, Kenya. (University of Nairobi, 2016).

[15] Nanyanzi M, Kizito E, Masanza M, Sseruwu G, Tenywa M (2018). Effect of different rates of poultry manure and bio-Slurry on the yield of Solanum aethiopicum Shum. Journal of Agricultural Science.

[16] Ssekabembe CK (2007). Comparison of research on sesame (Sesamum indicum) and nakati (Solanum aethiopicum) at Makerere University. African Crop Science Conference Proceedings.

[17] Sseremba, G. *et al*. Stability of Solanum aethiopicum Shum accessions under varied water deficit stress levels and identification of pertinent breeding traits for resistance to water shortage. *Euphytica* **214** (2018).

[18] Ssekabembe CK, Odong T (2008). Division of labour in nakati (Solanum aethiopicum) production in central Uganda. African Journal of Agricultural Research.

[19] Lipiec J, Doussan C, Nosalewicz A, Kondracka K (2013). Effect of drought and heat stresses on plant growth and yield: a review. International Agrophysics.

[20] Nakanwagi M, Sseremba G, Masanza M, Kizito E (2018). Performance of Solanum aethiopicum Shum group accessions under repetitive drought stress. Journal of Plant Breeding and Crop Science.

[21] Kabod N (2018). Stability for descriptors of Solanum aethiopicum Shum group (family Solanaceae). Journal of Plant Breeding and Crop Science.

[22] Sseremba G, Tongoona P, Eleblu JSY, Danquah EY, Kizito EB (2018). Linear discriminant analysis of structure within African eggplant ‘Shum’. African Crop Science Journal.

[23] Farooq, M., Hussain, M., Wahid, A. & Siddique, K. H. Drought stress in plants: an overview. In *Plant responses to drought stress*, 1–6 (Springer-Verlag, 2012).

[24] Banik P, Zeng W, Tai H, Bizimungu B, Tanino K (2016). Effects of drought acclimation on drought stress resistance in potato (Solanum tuberosum L.) genotypes. Environmental and Experimental Botany.

[25] Kesiime VE (2016). Characterization and Evaluation of Potato Genotypes (Solanum tuberosum L) for Tolerance to Drought in Uganda. American Journal of Potato Research.

[26] Osakabe, Y., Osakabe, K., Shinozaki, K. & Tran, L.-S. P. Response of plants to water stress. *Frontiers in Plant Science* **5** (2014). 10.3389/fpls.2014.00086 PMC3952189 24659993

[27] Fahad S (2017). Crop production under drought and heat stress: plant responses and management options. Frontiers in Plant Science.

[28] Kumar R, Solankey S, Singh M (2012). Breeding for drought tolerance in vegetables. Vegetable Science.

[29] Sseremba G, Tongoona P, Eleblu J, Danquah E, Kizito E (2018). Heritability of drought resistance in Solanum aethiopicum Shum group and combining ability of genotypes for drought tolerance and recovery. Scientia Horticulturae.

[30] Zhang X, Lei L, Lai J, Zhao H, Song W (2018). Effects of drought stress and water recovery on physiological responses and gene expression in maize seedlings. BMC Plant Biology.

[31] Gong L (2015). Transcriptome profiling of the potato (Solanum tuberosum L.) plant under drought stress and water-stimulus conditions. PLoS One.

[32] Yoshida T, Mogami J, Yamaguchi-Shinozaki K (2014). ABA-dependent and ABA-independent signaling in response to osmotic stress in plants. Current Opinion in Plant Biology.

[33] Gramazio, P. *et al*. Comparison of transcriptome-derived simple sequence repeat (SSR) and single nucleotide polymorphism (SNP) markers for genetic fingerprinting, diversity evaluation, and establishment of relationships in eggplants. *Euphytica* **213** (2017).

[34] Gramazio, P. *et al*. Transcriptome analysis and molecular marker discovery in Solanum incanum and S. aethiopicum, two close relatives of the common eggplant (Solanum melongena) with interest for breeding. *BMC Genomics* **17** (2016). 10.1186/s12864-016-2631-4 PMC4841963 27108408

[35] Blum A (2005). Drought resistance, water-use efficiency, and yield potential—are they compatible, dissonant, or mutually exclusive?. Australian Journal of Agricultural Research.

[36] Fang Y, Xiong L (2015). General mechanisms of drought response and their application in drought resistance improvement in plants. Cellular and Molecular Life Sciences.

[37] Galmés J (2013). Leaf responses to drought stress in Mediterranean accessions of Solanum lycopersicum: anatomical adaptations in relation to gas exchange parameters. Plant, Cell & Environment.

[38] Weijde TV (2017). Impact of drought stress on growth and quality of miscanthus for biofuel production. Global Change Biology Bioenergy.

[39] Blum A (2009). Effective use of water (EUW) and not water-use efficiency (WUE) is the target of crop yield improvement under drought stress. Field Crops Research.

